# Exosomal Long Interspersed Nuclear Element‐1 Analytes Discriminate Histologic Subtypes, Sex, and Clinicopathological Characteristics of Patients with Non‐Small Cell Lung Cancer

**DOI:** 10.1002/mco2.70472

**Published:** 2025-11-10

**Authors:** Abeer A. I. Hassanin, Kenneth S. Ramos

**Affiliations:** ^1^ Center For Genomic and Precision Medicine Texas ARM Institute of Biosciences and Technology Texas Medical Center Houston Texas USA; ^2^ Department of Animal Wealth Development Faculty of Veterinary Medicine Suez Canal University Ismailia Egypt

**Keywords:** diagnostic biomarker, LINE‐1, non‐small cell lung cancers, plasma exosomes

## Abstract

Lung cancer is the leading cause of cancer‐related mortality worldwide, with lung squamous cell carcinoma (LUSC) and lung adenocarcinoma (LUAD) representing the most common non‐small cell lung cancer (NSCLC) subtypes. The invasive procedures typically required to obtain specimens for clinical evaluation pose significant risks and can delay patient care. To address these limitations, analysis of cancer‐related biomarkers in circulating exosomes has emerged as a promising liquid biopsy approach. In this study, levels of long interspersed nuclear element‐1 (LINE‐1) mRNA were measured in ostensibly healthy controls and compared with those in patients with LUSC and LUAD. Both LINE‐1 ORF1 and ORF2 mRNA were readily detectable across all cancer stages in both female and male patients, with expression patterns correlating with histologic subtype, tumor stage, tumor size, lymph node involvement, distant metastasis, and smoking status. Receiver operating characteristic analyses confirmed the robustness of this approach in distinguishing NSCLC subtypes and associated clinicopathological features. Collectively, these findings highlight exosomal LINE‐1 mRNA as a readily accessible biomarker for precision profiling of NSCLC. The strong diagnostic and prognostic performance of this liquid biopsy platform underscores its potential to advance the clinical management of patients with NSCLC.

## Graphical abstract

Exosomal long interspersed nuclear element‐1 (LINE‐1) analytes exhibit distinct expression profiles across histologic subtypes of non‐small cell lung cancer (NSCLC) and demonstrate significant associations with clinicopathological characteristics. These findings underscore the potential of exosomal LINE‐1 mRNA as a minimally invasive biomarker for NSCLC stratification, prognostic evaluation, and personalized therapeutic management.

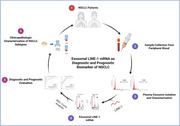

## 1 Introduction

Lung cancer ranks as the second most common cause of new cancer cases in both males and females in the United States and the second‐leading cause of cancer‐related deaths in females worldwide [1, 2]. The predominant lung cancer subtypes are lung squamous cell carcinoma (LUSC) and lung adenocarcinoma (LUAD), which together represent most non‐small cell lung cancers (NSCLC) [3, 4]. Recent investigations have indicated that LUSC and LUAD should be categorized and managed as distinct forms of cancer [5]. To this end, precision oncology has increasingly focused on the individualization of cancer diagnosis and treatment, and importantly, actionable risk stratifiers of malignancy. For lung cancer patients, the invasive procedures required to procure specimens for clinical evaluation can pose significant risks and create detrimental delays in care. To overcome these limitations, measurements of cancer analytes in extracellular vesicles(EVs) in blood have been proposed as molecular diagnostics of lung cancer [6, 7].

To date, extracellular vesicles (EVs) have been shown to include different proteins, RNAs, and DNA fragments [8]. The molecular composition of EVs reflects the intracellular condition of their source cells; a growing amount of data suggests that the intracellular state of cancer‐derived EV loads is strikingly comparable to that of their parental cells [9]. Consequently, EV‐based liquid biopsy can lead to the early detection of cancer, the monitoring of cancer pathological states, and the selection of therapy based on the disease's biology and projected treatment response [10].

Exosomes are membrane vesicles with a size range of 30–150 nm that are produced by several kinds of cells in the body [11, 12, 13] from the limiting membrane of late endosomes by invagination and budding [14, 15]. They concentrate in cytosolic multivesicular bodies, from which they are released by membrane fusion [14, 15]. Vesicle release is a very active mechanism in growing cells, such as cancer cells [16], their composition may resemble that of the cell of origin, creating a novel and accessible source for tumor profiling. Of interest is that the mRNA and proteins encoded by the oncogenic long interspersed nuclear element‐1 (LINE‐1) retrotransposon are present in circulating EVs of NSCLC patients [7, 17], and that exosomes isolated from ostensibly healthy individuals and NSCLC patients are retrotransposition competent and can serve as a source for de novo insertions in tumors [7, 18, 19, 20].

LINE‐1 retrotransposons are transposable genetic elements that mobilize to new genomic locations via RNA intermediates. LINE‐1 has an internal promoter within its 5′ untranslated region (5′ UTR), two open reading frames (ORFs) that encode ORF1p and ORF2p, and a 3′ UTR with a poly (A) tail [21]. Both ORFs are transcribed from a shared 5′ UTR promoter by RNA polII and translated during a single retrotransposition cycle. Although LINE‐1 retroelements are readily silenced via epigenetic and posttranscriptional mechanisms in somatic cells [22], several cancers, including lung cancers, undergo active and repeated cycles of retrotransposition [23, 24]. As such, substantial linkages between LINE‐1, cancer status, tumor instability, and cancer mortality have been established [25, 26, 27, 28, 29]. We recently introduced proof‐of‐principle evidence that the LINE‐1 status in tumor cells and lung cancer tissues mirrors the LINE‐1 cargo in their corresponding EVs [30]. Because the number of circulating exosomes increases in the body fluids of individuals with lung cancer [31], these particles provide a readily accessible source of lung tumor material for precision‐based tumor profiling and prognostic evaluation of patients with NSCLC.

Despite these intriguing relationships, our present knowledge is insufficient to assess the utility of EV‐associated LINE‐1 analytes as liquid biopsies for lung cancer and to systematically evaluate sex‐ and subtype‐specific differences among NSCLC patients. To fill these gaps, the present studies were conducted to evaluate the usefulness of exosome‐derived LINE‐1 ORF1 and ORF2 mRNA profiles as diagnostic and prognostic biomarkers of NSCLC. Our analysis emphasized sex and clinicopathologic differences.

## 2 Results

### 2.1 Characterization of Plasma‐Derived Exosomes

**FIGURE 1:**
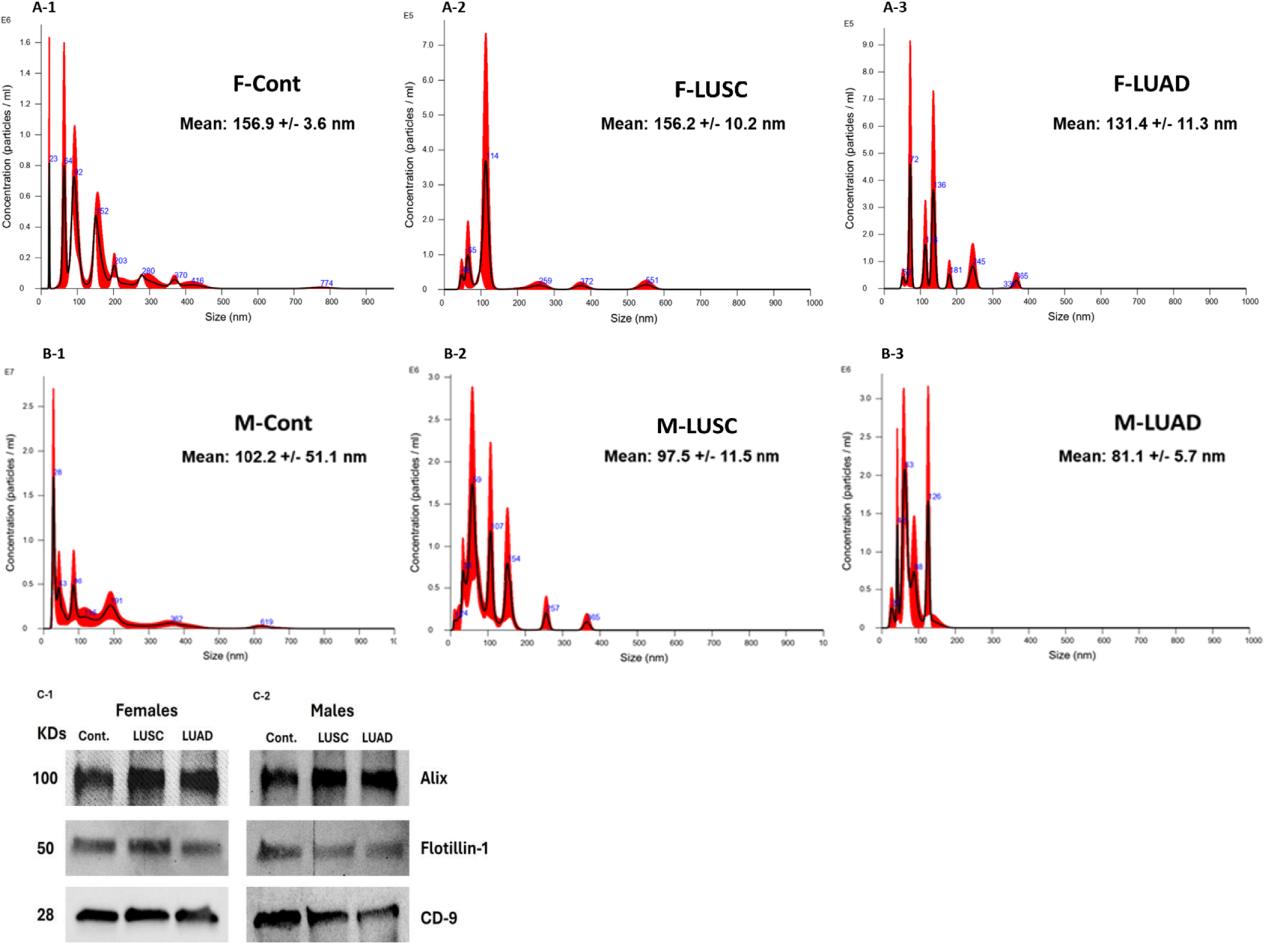
Characterization of plasma‐derived exosomes; (A1–A3) Representative NTA distribution of plasma exosomes in females: (A1) control group; (A2) LUSC patients; (A3) LUAD patients. (B1–B3) Representative NTA distribution of plasma exosomes in males: (B1) control group; (B2) LUSC patients; (B3) LUAD patients. (C1 and C2) Western blot analysis of exosomal protein markers (Alix, Flotillin‐1, CD‐9) in both sexes.

Plasma exosomes used in all experiments were extracted from 1 mL of plasma. The average yield varied among samples, with an average of 5.94 × 10^7^ in the control (Cont.) group, 4.07 × 10^7^ in the LUSC group, and 5.45 × 10^7^ in the LUAD group. Exosomes were characterized using the NanoSight NS300 (NTA). The average diameters of exosomes isolated from healthy female Cont., LUSC patients, and LUAD patients were 156.9 ± 3.6, 156.2 ± 10.2, and 131.4 ± 11.3 nm, respectively (Figure 1A1–A3). Smaller sizes were measured for males, with average exosome diameters for healthy male Cont., LUSC patients, and LUAD patients of 102.2 ± 51.1, 97.5 ± 11.5, and 81.1 ± 5.7 nm, respectively (Figure 1B1–B3). Western blot analysis confirmed the presence of the conventional exosomal protein markers, Alix, Flotillin‐1, and CD‐9, in both female and male exosomes (Figure 1C1,C2).

### 2.2 Exosomal Profiles of LINE‐1 ORF1 and ORF2 mRNAs in NSCLC

**FIGURE 2:**
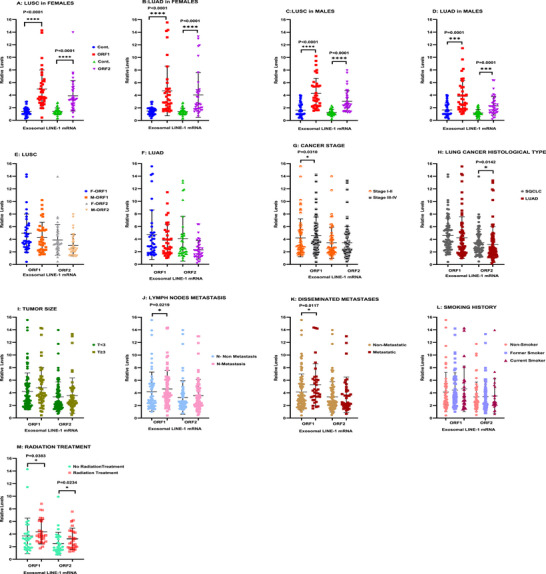
(A–M) Exosomal LINE‐1 ORF1 and ORF2 mRNAs in female and male patients with LUSC and LUAD. (A) Levels of LINE‐1 ORF1 and ORF2 mRNAs were significantly elevated in LUSC female patients (*n* = 31) compared with controls (*n* = 31), with higher levels of ORF1 mRNA than ORF2 mRNA. (B) LINE‐1 ORF1 and ORF2 mRNA levels were significantly higher in female LUAD patients (*n* = 31) compared with controls (*n* = 31). (C) Both LINE‐1 ORF1 and ORF2 mRNAs were significantly higher in males with LUSC (*n* = 31) compared with controls (*n* = 31). (D) Highly significant variations in the levels of LINE‐1 ORF1 and ORF2 mRNAs were seen in LUAD male patients (*n* = 31) compared with controls (*n* = 31). (E) There were no significant differences in LINE‐1 ORF1 and ORF2 mRNA levels between female (*n* = 31) and male (*n* = 31) LUSC patients. (F) No significant differences were observed in LINE‐1 ORF1 and ORF2 mRNA levels between female (*n* = 31) and male (*n* = 31) LUAD patients. (G–M) Correlation between LINE‐1 ORF1 and ORF2 mRNA levels and clinicopathological characters. (G) Patients with NSCLC Stages III and IV (*n* = 60) showed significantly higher levels of LINE‐1 ORF1 mRNA compared with Stages I and II (*n* = 56). (H) Significantly higher levels of LINE‐1 ORF2 were seen in patients with LUSC (*n* = 62) compared with LUAD (*n* = 62). (I) LINE‐1 ORF1 and ORF2 levels were slightly higher in larger sized tumors (*T* ≥ 3) (*n* = 34) compared with smaller tumors (*T* < 3) (*n* = 82). (J) Significantly higher levels of LINE‐1 ORF1 correlated with lymph node metastases (*n* = 118). (K) The level of LINE‐1 ORF1 mRNA was significantly higher in NSCLC metastatic tumors (*n* = 27) compared with nonmetastatic (*n* = 91). (L) Current smokers had slightly higher levels of both LINE‐1 ORF1 and ORF2 mRNAs (*n* = 20) compared with former (*n* = 51) or nonsmokers (*n* = 52). (M) Exosomal LINE‐1 ORF1 and ORF2 mRNA levels were significantly increased after radiation therapy (*n* = 39). Unpaired *t*‐test was applied. *p < 0.05, **p < 0.005, ***p < 0.001, ****p < 0.0001. Horizontal bars indicate mean values.

Measurements were completed across all NSCLC grades, stages, and ages in females and males to examine sex‐specific differences and to discriminate clinicopathologic characteristics. In females with LUSC and LUAD, both ORF1 and ORF2 mRNA levels were significantly elevated compared with Cont. (*p* < 0.0001) (Figure 2A,B, respectively). Comparable patterns were seen in males with LUSC (*p* < 0.0001) (Figure 2C) and LUAD (*p* = 0.0001) (Figure 2D). The response profiles of females and males were comparable, as no significant differences between them were found (Figure 2E,F, respectively). The levels of exosomal ORF1 mRNA in NSCLC patients at Stages III and IV were significantly higher than at Stages I and II (*p* = 0.0310) (Figure 2G). LUSC patients had significantly elevated levels of exosomal ORF2 mRNA compared with LUAD patients (*p* = 0.0142) (Figure 2H). Patients with larger tumors (*T* ≥ 3) showed slightly nonsignificant higher levels of both ORF1 and ORF2 mRNAs compared with those with smaller tumors (*T* < 3) (Figure 2I). Patients with lymph nodes metastases and disseminated metastases tumors exhibited significantly higher ORF1 mRNA levels (*p* = 0.0219 and *p* = 0.0117, respectively) compared with those with nonmetastatic tumors. (Figures 2J,K). Current or former smoking status increased LINE‐1 ORF1 mRNA levels in some subjects, but these differences were not significant (Figure 1L). The history of radiation therapy significantly increased both ORF1 and ORF2 mRNA levels with *p* = 0.0383 for ORF1 and *p* = 0.0234 for ORF2 (Figure 2M). Together these data indicate that exosomal LINE‐1 mRNAs are readily detectable in both females and males with NSCLC and discriminate histologic subtypes and clinicopathological characteristics.

### 2.3 Diagnostic and Prognostic Utility of Exosomal LINE‐1 mRNAs in the Training Cohort

**FIGURE 3:**
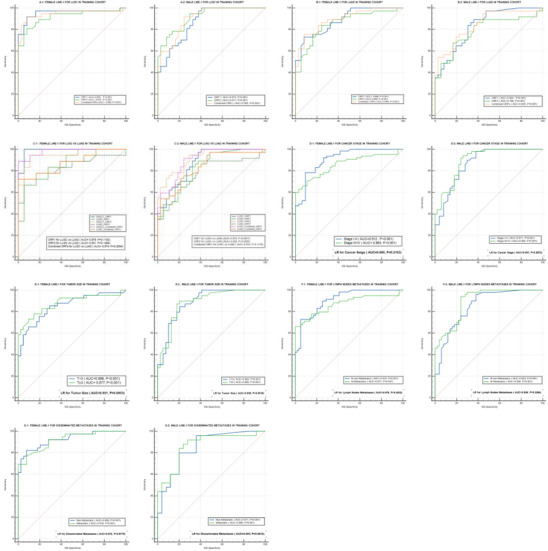
Diagnostic value of exosomal LINE‐1 mRNAs in the training cohort distinguishing. (A1 and A2) LUSC patients compared with ostensibly healthy controls, in females, the LINE‐1 ORF1 and ORF2 mRNAs showed superior diagnostic performance distinguishing LUSC patients (*n* = 31) from healthy controls (*n* = 31) (AUC values for LINE‐1 ORF1 = 0.955, for LINE‐1 ORF2 = 0.919; for both ORFs = 0.948). In males LINE‐1 ORF2 and both ORFs showed a superior diagnostic power (AUC for LINE‐1 ORF2 = 0.917, and for both AUC = 0.905), ORF1 showed excellent diagnostic power (AUC = 0.873) distinguishing LUSC patients (*n* = 31) from controls (*n* = 31). (B1 and B2) LUAD patients compared with ostensibly healthy controls. In females, the diagnostic performance of LINE‐1 ORF1, LINE‐1 ORF2, and both ORFs together was excellent in differentiating LUAD patients (*n* = 31) from healthy controls (*n* = 31) (AUC values for LINE‐1 ORF1 = 0.886, for LINE‐1 ORF2 = 0.885, for both ORFs = 0.894). In males, LINE‐1 ORF1 and both ORFs had an excellent diagnostic value (AUC for LINE‐1 ORF1 = 0.824, AUC for both ORFs = 0.7845), while LINE‐1 ORF2 showed acceptable diagnostic values (AUC = 0.786). (C1 and C2) Contrasting LUSC versus LUAD patients. In females, LINE‐1 ORF1 and ORF2 alone or in combination showed superior diagnostic capabilities distinguishing LUSC from LUAD (AUC for LINE‐1 ORF1 = 0.979, LINE‐1 ORF2 was 0.931, and both ORFs = 0.974). In males, LINE ORF2 and both ORFs showed superior diagnostic performance (AUC for LINE‐1 ORF2 = 0.932; both ORFs = 0.915), while LINE‐1 ORF1 exhibited excellent diagnostic performance distinguishing between the two histological subtypes (AUC = 0.873). (D1 and D2) Cancer stage. The AUC values for female LINE‐1 were 0.912, 0.863, and 0.980 for distinguishing early (Stages I–II) and late (Stages III–IV) cancer stages from healthy controls, and for distinguishing between the stages, respectively. In comparison, the AUC values for male LINE‐1 were 0.871, 0.894, and 0.957. (E1 and E2) Tumor size. The AUC values for female LINE‐1 were 0.886, 0.877, and 0.951 for differentiating small (*T* < 3) and large (*T* ≥ 3) sized tumors from healthy controls, and for differentiating tumors based on size, respectively. By comparison, the AUC values for male LINE‐1 were 0.883, 0.885, and 0.939. (F1 and F2) Lymph nodes metastasis. The AUC values for female LINE‐1 were 0.910, 0.877, and 0.978 for differentiating tumors without and with lymph nodes metastasis from healthy controls, and for differentiating tumors depending on the presence or absence of lymph node metastases, respectively, in contrast, male LINE‐1 had AUC values of 0.853, 0.892, and 0.936. (G1 and G2) Disseminated metastases. The AUC values for female LINE‐1 were 0.895, 0.919, and 0.974 for distinguishing nonmetastatic and metastatic cancers from healthy controls, and for distinguishing between nonmetastatic and metastatic tumors, respectively. On the other hand, the male LINE‐1 exhibited AUC values of 0.871, 0.866, and 0.965.

Receptor operator characteristic (ROC) analyses were used to evaluate and compare the diagnostic utility of LINE‐1 mRNAs in patients with LUSC and LUAD (Figure 3A–C). Our classification model was trained using the first set of cohort values. The area under curve (AUC), sensitivity (SEN), specificity (SPE), and all cut‐off values were all determined using ROC analysis and summarized in Table 1. In females (Figure 3A1), ORF1 mRNA distinguished LUSC cases from Cont. with an AUC of 0.955. ORF2 mRNA also demonstrated a high level of accuracy with an AUC of 0.919. Together, ORF1 and ORF2 mRNA levels demonstrated superior diagnostic utility, with an AUC of 0.948 differentiating LUSC cases from Cont. In males (Figure 3A2), ORF1 mRNA differentiated LUSC cases from Cont., with an AUC of 0.873, ORF2 mRNA differentiated LUSC male cases from Cont. with an AUC of 0.917, and the combination of both mRNAs differentiated LUSC male cases from Cont., with an AUC of 0.905.

**TABLE 1:** Diagnostic performance of LINE‐1 mRNAs in the training and validation cohorts.

			Training cohort				Validation cohort			
Sex	NSCLC subtype	LINE‐1	AUC (95% CI)	SEN	SPE	Criterion	AUC (95% CI)	SEN	SPE	Criterion
Female	LUSC	ORF1	0.955 (0.879–0.989)	91.9	91.9	>2.09	0.925 (0.828–0.977)	80.0	96.8	>2.69
		ORF2	0.919 (0.831–0.969)	81.1	91.9	>2.25	0.915 (0.815–0.971)	76.7	96.8	>2.78
		Combined	0.948 (0.870–0.986)	91.9	91.9	>2.04	0.923 (0.826–0.976)	80.0	93.5	>2.62
	LUAD	ORF1	0.886 (0.791–0.948)	73.0	91.9	>2.09	0.885 (0.779–0.952)	90.3	83.9	>3.05
		ORF2	0.885 (0.754–0.926)	83.8	75.7	>1.61	0.886 (0.780–0.953)	90.3	77.4	>2.61
		Combined	0.894 (0.801–0.954)	75.7	91.9	>2.04	0.904 (0.802–0.964)	77.4	96.8	>3.165
	LUSC vs. LUAD	ORF1	0.979 (0.915–0.998)	−	−	−	0.966 (0.884–0.995)	−	−	−
		ORF2	0.931 (0.847–0.977)	−	−	−	0.969 (0.889–0.996)	−	−	−
		Combined	0.974 (0.907–0.997)	−	−	−	0.973 (0.895–0.998)	−	−	−
Male	LUSC	ORF1	0.873 (0.775–0.939)	100	59.5	>1.49	0.861 (0.750–0.936)	71.0	90.3	>1.99
		ORF2	0.917 (0.830–0.969)	94.6	70.3	>1.39	0.903 (0.801–0.964)	87.1	83.9	>1.85
		Combined	0.905 (0.814–0.961)	91.9	75.7	>1.8	0.918 (0.820–0.972)	90.3	83.9	>1.72
	LUAD	ORF1	0.824 (0.718–0.902)	89.2	62.2	>1.55	0.898 (0.794–0.960)	77.4	90.3	>1.99
		ORF2	0.786 (0.675–0.873)	67.6	83.8	>1.49	0.883 (0.776–0.951)	74.2	90.3	>1.92
		Combined	0.845 (0.742–0.919)	73.0	80.6	>1.88	0.932 (0.839–0.981)	90.3	83.9	>1.86
	LUSC vs. LUAD	ORF1	0.873 (0.775–0.939)	−	−	−	0.933 (0.840–0.981)	−	−	−
		ORF2	0.932 (0.849–0.977)	−	−	−	0.946 (0.857–0.987)	−	−	−
		Combined	0.915 (0.827–0.967)	−	−	−	0.961 (0.879–0.994)	−	−	−

Abbreviations: AUC, area under the curve; LUSC, lung squamous cell cancer; LUAD, lung adenocarcinoma; NSCLC, non‐small cell lung cancer; SEN, sensitivity; SPE, specificity.

In females with LUAD (Figure 3B1), ORF1 mRNA showed an AUC of 0.886 distinguishing cases from Cont., while ORF2 mRNA showed an AUC of 0.885, and the combined mRNAs had an AUC of 0.894. In males with LUAD (Figure 3B2), ORF1 mRNA distinguished cases from Cont. with an AUC of 0.824, while ORF2 mRNA had an AUC of 0.786. The combined mRNAs in male cases had an AUC of 0.845 distinguishing cases from Cont.

Figures 3C shows that ROC analysis efficiently distinguished patients with LUSC from those with LUAD. In females (Figure 3C1), ORF1 mRNA had an AUC of 0.979 (*p* = 0.1192), while ORF2 mRNA had an AUC of 0.931 (*p* = 0.1466). When the mRNAs were combined the AUC was 0.974 (*p* = 0.2054). In males (Figure 3C2), ORF1 mRNA exhibited an AUC of 0.873 (*p* = 0.0917) differentiating LUSC from LUAD patients, while ORF2 mRNA had an AUC of 0.932 (*p* = 0.0252). When the mRNAs were combined, the AUC was 0.915 (*p* = 0.1179). Collectively, these data establish the exceptional diagnostic power of exosomal LINE‐1 mRNAs distinguishing between LUSC and LUAD patients and Cont., with higher AUC values in female patients compared with male counterparts. Further, these results suggest that the classifier may be an innovative predictive tool of high accuracy and potential clinical significance.

Additional analyses examined the prognostic performance of LINE‐1 mRNA differentiating clinicopathological characteristics (cancer stage, tumor size, lymph nodes metastasis, and disseminated metastases) among NSCLC patients (Figure 3D,G). The AUC, SEN, SPE, and all cut‐off values were determined by ROC analysis and summarized in Table 2. Female LINE‐1 mRNA showed an AUC of 0.912 distinguishing early‐stage NSCLC patients (Stages I–II) from Cont. and an AUC of 0.863 distinguishing late‐stage NSCLC patients (Stages III–IV) from Cont., and an AUC of 0.980 differentiating between early‐stage and late‐stage NSCLC patients (Figure 3D1). In males, AUC values were 0.871, 0.894, and 0.957 distinguishing early‐stage or late‐stage NSCLC patients from Cont. and between early‐stage and late‐stage NSCLC, respectively (Figure 3D2). Female LINE‐1 mRNA had an AUC of 0.886 differentiating patients with small tumors (*T* < 3) from Cont. compared with 0.877 for those with large tumors (*T* ≥ 3) and 0.951 differentiating between small (*T* < 3) and large (*T* ≥ 3) tumors (Figure 3E1). Comparable analyses for males showed AUC values of 0.883, 0.885, and 0.939, respectively (Figure 3E2). Female LINE‐1 mRNA had an AUC of 0.910 and 0.877 distinguishing patients without lymph node metastasis or with lymph node metastasis from Cont., respectively, and 0.978 differentiating between those without and with lymph node metastases (Figure 3F1). Comparable analyses for males showed AUCs of 0.853, 0.892, and 0.936, respectively (Figure 3F2). Analyses for disseminated metastasis showed an AUC of 0.895 for the ability of female LINE‐1 mRNA to distinguish patients with nonmetastatic tumors from Cont. (Figure 3G1). The AUC differentiating patients with metastatic tumors from Cont. was 0.919 and 0.974 for patients with nonmetastatic versus metastatic tumors. In males, the comparable AUC values were 0.871, 0.866, and 0.965, respectively (Figure 3G2)

**TABLE 2:** Prognostic performance of LINE‐1 mRNA in the training and validation cohorts.

Sex	TNM staging		Training cohort				Validation cohort			
			AUC (95% CI)	SEN	SPE	Criterion	AUC (95% CI)	SEN	SPE	Criterion
Female	Stage	I–II	0.912 (0.849–0.954)	78.5	90.8	>2.09	0.871 (0.785–0.932)	78.3	89.1	>2.78
		III–IV	0.863 (0.792–0.916)	71.2	89.4	>1.99	0.896 (0.829–0.943)	88.7	78.1	>2.69
		I–II vs. III–IV	0.980 (0.938–0.996)	−	−	−	0.957 (0.894–0.989)	−	−	−
	Tumor size (T)	*T *< 3	0.886 (0.829–0.929)	73.9	89.8	>2.09	0.874 (0.817–0.919)	81.8	80.0	>2.64
		*T* ≥ 3	0.877 (0.786–0.939)	78	85.4	>1.93	0.962 (0.849–0.997)	90.0	90.0	>2.82
		*T* < 3 vs. *T* ≥ 3	0.951 (0.880–0.987)	−	−	−	0.998 (0.907–1.000)	−	−	−
	Lymph node involvement (N)	No	0.910 (0.843–0.955)	72.9	94.9	>2.09	0.854 (0.748–0.928)	73.5	94.1	>3.05
		Yes	0.877 (0.813–0.926)	64.4	100	>2.96	0.906 (0.847–0.947)	86.5	78.9	>2.64
		No vs. Yes	0.978 (0.933–0.996)	−	−	−	0.959 (0.881–0.992)	−	−	−
	Disseminated metastasis (M)	No	0.895 (0.841–0.935)	76.9	89.0	>2.09	0.905 (0.846–0.947)	78.4	90.8	>2.78
		Yes	0.919 (0.835–0.969)	76.9	92.3	>2.37	0.871 (0.764–0.942)	81.2	84.4	>3.06
		No vs. Yes	0.974 (0.910–0.997)	−	−	−	0.954 (0.870–0.991)	−	−	−
Male	Cancer stage	I–II	0.871 (0.789–0.930)	98.0	62.0	>1.39	0.917 (0.843–0.964)	95.8	75.0	>1.53
		III–IV	0.894 (0.832–0.940)	93.0	71.8	>1.66	0.853 (0.784–0.907)	68.6	88.6	>1.99
		I–II vs. III–IV	0.957 (0.897–0.988)	−	−	−	0.957 (0.896–0.988)	−	−	−
	Tumor size (T)	*T* < 3	0.883 (0.823–0.928)	90.0	71.2	>1.49	0.914 (0.855–0.955)	81.4	88.6	>1.93
		*T* ≥ 3	0.885 (0.792–0.946)	89.7	79.5	>1.89	0.854 (0.760–0.922)	92.9	66.7	>1.55
		*T* < 3 vs. *T* ≥ 3	0.939 (0.861–0.981)	−	−	−	0.926 (0.848–0.972)	−	−	−
	Lymph node involvement (N)	No	0.853 (0.768–0.916)	96.0	62.0	>1.39	0.897 (0.809–0.954)	85.0	87.5	>1.99
		Yes	0.892 (0.828–0.938)	94.2	71.0	>1.55	0.887 (0.823–0.933)	91.7	72.2	>1.55
		No vs. Yes	0.936 (0.869–0.975)	−	−	−	0.971 (0.907–0.996)	−	−	−
	Disseminated metastasis (M)	No	0.871 (0.816–0.914)	93.9	64.3	>1.39	0.899 (0.844–0.940)	79.1	88.4	>1.99
		Yes	0.866 (0.739–0.945)	92.0	72.0	>1.42	0.854 (0.729–0.937)	96.2	73.1	>1.55
		No vs. Yes	0.965 (0.870–0.997)	−	−	−	0.981 (0.897–1.000)	−	−	−

Abbreviations: AUC, area under the curve; LUSC, lung squamous cell cancer; LUAD, lung adenocarcinoma; NSCLC, non‐small cell lung cancer; SEN, sensitivity; SPE, specificity.

### 2.4 Validation of ROC Curve Findings

**FIGURE 4:**
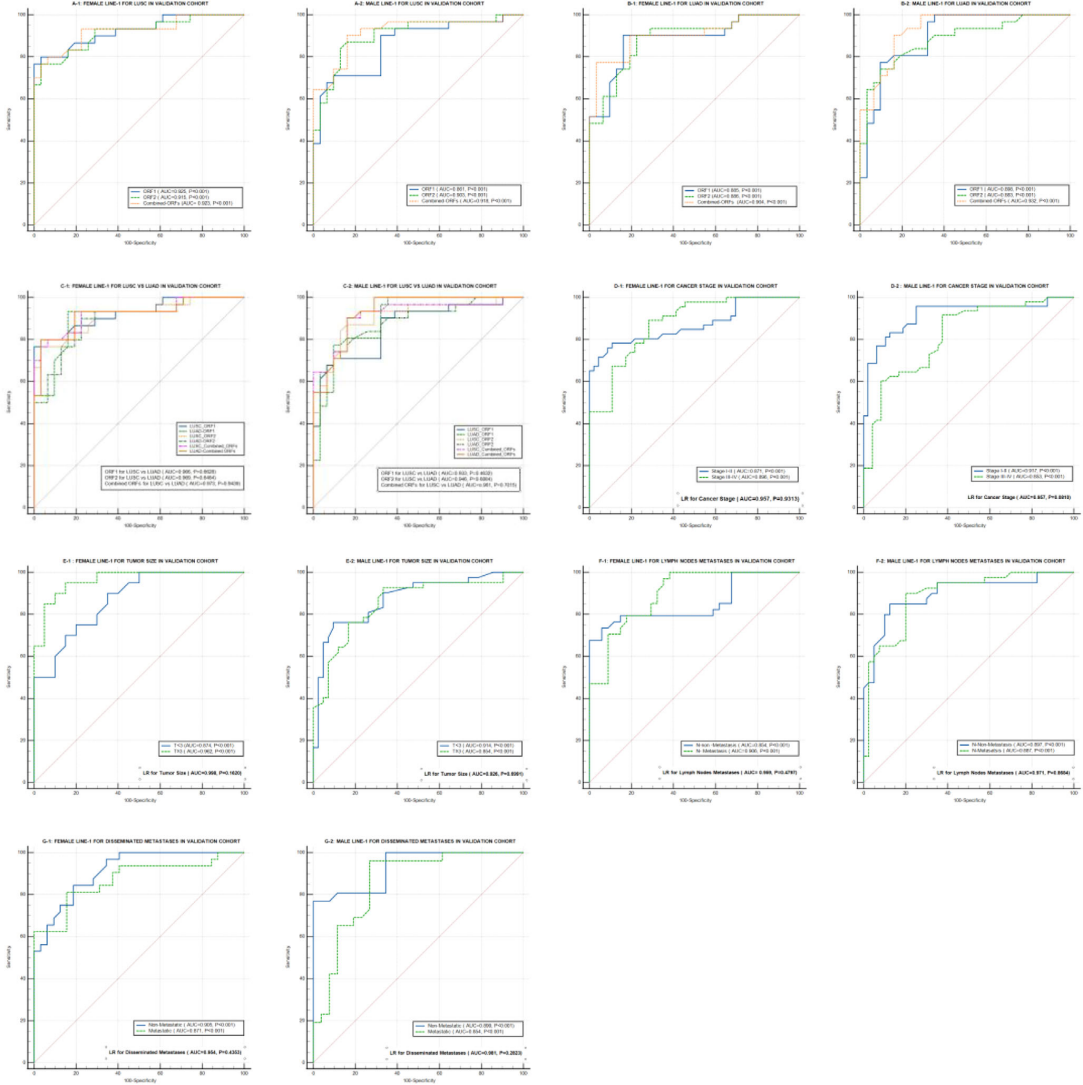
Diagnostic value of exosomal LINE‐1 mRNAs in the validation cohort distinguishing. (A1 and A2) LUSC patients (*n* = 19) compared with ostensibly healthy controls (*n* = 19). (B1 and B2) LUAD patients (*n* = 19) compared with ostensibly healthy controls (*n* = 19). (C1 and C2) Contrasting LUSC (*n* = 19) versus LUAD patients (*n* = 19). (D1 and D2) Cancer stage (*n* = 38). (E1 and E2) Tumor size (*n* = 38). (F1 and F2) Lymph nodes metastasis (*n* = 38). (G1 and G2) Disseminated metastases (*n* = 38).

To ensure the robustness and generalizability of the findings, an independent validation set was examined (Figure 4A–G). The AUC, SEN, SPE, and all cut‐off value are presented in Tables 1 and 2. The validation cohort showed AUCs of 0.925, 0.915, and 0.923 differentiating between LUSC patients and Cont. using female LINE‐1 ORF1 mRNA, ORF2 mRNA, or both, respectively (Figure 4A1). The AUCs for male LINE‐1 mRNAs were 0.861, 0.903, and 0.918 (Figure 4A2). The AUCs discriminating between female LUAD patients and Cont. were 0.885, 0.886, and 0.904, respectively (Figure 4B1). In comparison, the AUCs for male LINE‐1 mRNA were 0.898, 0.883, and 0.932, respectively (Figure 4B2). Female LINE‐1 ORF1 mRNA, ORF2 mRNA, and both had AUC values of 0.966, 0.969, and 0.973, respectively, distinguishing between the two histological subtypes (Figure 4C1). Male LINE‐1 AUC values were 0.933, 0.946, and 0.961, respectively (Figure 4C2). Female LINE‐1 mRNAs discriminated between early‐stage tumors, late‐stage tumors, and healthy Cont., and between early and late stage, with AUC values of 0.871, 0.896, and 0.957, respectively (Figure 4D1), compared with males with AUC values of 0.917, 0.853, and 0.957, respectively (Figure 4D2). Regarding tumor size, female LINE‐1 mRNAs effectively differentiated between small tumors, large tumors, and Cont. and smaller versus larger tumor sizes with AUC values of 0.874, 0.962, and 0.998, respectively (Figure 4E1). Males showed AUC values of 0.914, 0.854, and 0.926, respectively (Figure 4E2). Female LINE‐1 mRNAs distinguished tumors without and with lymph node metastasis from Cont., with AUC values of 0.854, 0.906, and 0.959, respectively (Figure 4F1). Males showed AUC values of 0.897, 0.887, and 0.971, respectively (Figure 4F2). The classification model differentiated nonmetastatic tumors, metastatic tumors, and Cont., as well as nonmetastatic and metastatic tumors in females, with AUC values of 0.905, 0.871, and 0.954, respectively (Figure 4G1). Males showed AUC values of 0.899, 0.854, and 0.981, respectively (Figure 4G2). The fidelity of our validation cohort findings confirmed the efficacy and reliability of our classification model discriminating NSCLC histological subtypes and their clinicopathological characteristics in an independent patient population.

## 3 Discussion

Evidence is presented here that LINE‐1 mRNA cargos in plasma‐derived exosomes can be used as diagnostic and prognostic indicators in the clinical evaluation of patients with NSCLC. The use of plasma exosomes overcomes many of the limitations of tissue derived biopsies, that while considered the gold standard for cancer diagnosis, require surgical removal of tissue, fail to capture the full heterogeneity of the tumor, take days/weeks to process, and require specialized skills for both procurement and pathological evaluation. In sharp contrast, exosomal liquid biopsy methodology is less invasive and easy to process, allows for real‐time monitoring of disease dynamics, treatment response, and tracking of disease progression. Previous studies have shown that tumors release large quantities of exosomes into the general circulation, with the majority of circulating exosomes isolated in plasma originating from tumor tissue [32, 33]. Our prior work supports this, demonstrating that exosomal LINE‐1 analytes secreted by cultured tumor cells mirror those found in circulating exosomes from lung cancer patients [7]. Together, these findings establish the biological relevance of plasma‐based exosomal analyses and justify the use of circulating exosomal LINE‐1 mRNA as a surrogate for tissue‐derived LINE‐1 expression.

The classification model used was developed using independent training and validation sets to discriminate patients based on tumor histology (LUSC vs. LUAD), sex (female vs. males), and clinicopathological characteristics (cancer stage, tumor size, metastatic status). As such, the model holds considerable promise given the increasing demand for early identification and evaluation of patients at risk of lung malignancy. Despite major advances in precision‐based lung cancer diagnosis and treatment, the prevalence of solitary pulmonary nodules (SPN) continues to rise due to widespread, albeit inconsistent, use of low dose computed tomography screening. Various guidelines for SPN evaluation are available, but their utility remains challenging due to lack of specificity and consistency identifying patients at risk and limitations in the radiographic assessment of nodules [34]. As such, many patients enter a diagnostic odyssey that requires routine follow‐up and invasive interventions. Tissue biopsies have inherent drawbacks including their invasive nature, inaccessibility of tumors, insufficient tissue material, and limitations capturing tumor heterogeneity [35]. Given these limitations, liquid biopsies of NSCLC have garnered increasing attention [36]. Liquid biopsies can be used to risk stratify patients diagnosed with NSCLC and to monitor clinical trajectories. Exosomal LINE‐1 analytes released by tumor cells provide a noninvasive molecular signature of tumor activity and therefore, plasma measurements will likely become highly complementary to imaging, improving differentiation between malignant and benign nodules and enhancing early risk stratification.

A prominent role of LINE‐1 in lung oncogenesis was established in studies showing that hypomethylation of LINE‐1 occurs early in the disease and is associated with genetic instability and cancer development [37]. Hypomethylation has also been linked to high‐grade malignancy and poor prognosis in LUAD [38]. Others have shown that the formation of chimeric transcripts of LINE‐1 via somatic retrotransposition or antisense promoter activation is involved in metabolic reprogramming of lung cancer [39]. Direct evidence linking LINE‐1 to lung oncogenesis came from studies showing that forced expression of LINE‐1 in nontransformed human lung epithelial cells induces epithelial‐to‐mesenchymal transition, activates oncogenic programming, and induces malignant phenotypes [40, 41]. Somatic LINE‐1 retrotransposition has been linked to the onset and progression of LUSC [42]. We recently described the occurrence of LINE‐1 cargo exportation in plasma exosomes of ostensibly healthy subjects and cancer patients and showed that exosomal contents resemble the cells of origin and remain retrotransposition competence [7, 30].

In this study, we show that LINE‐1 analytes are present in plasma exosomes of NSCLC patients at all cancer stages, including metastatic tumors, and that measurements of these analytes can be useful as minimally invasive biomarkers of NSCLC to evaluate histologic, sex, and clinicopathologic profiles. ROC curve analyses of exosomal LINE‐1 mRNAs revealed high diagnostic and prognostic utility differentiating LUSC and LUAD cases from Cont., with stronger discriminating power for females. The classification was stronger for patients with LUSC compared with LUAD, with AUC values in the training cohort ranging from 0.948 to 0.955 for females and 0.873 to 0.917 for males and AUC values ranging from 0.915 to 0.925 for females and 0.861 to 0.918 for males in the validation cohort. These findings are consistent with the findings that Caucasian Americans with LUSC had greater numbers of LINE‐1 ORF1p+ cells in their tumors than patients with LUAD [43]. Exosomal LINE‐1 was higher in larger tumors with high metastatic burden and advanced TNM stage. While aggregate analyses established with confidence the high performance of LINE‐1 analytes in discriminating NSCLC from Cont., considerable dispersion of values was seen among lung cancer patients, especially those with LUAD. It is worth noting that the dispersion of values across the different endpoints examined was random, with high values preferentially seen for late stage and metastatic patients. Future assessment of polymorphic LINE‐1 variants and segregation of exosomes by tissue of origin may help to define patient‐to‐patient variations and clinical trajectories and outcomes.

Accurate pathological staging forms the cornerstone of personalized treatment planning, ensuring that patients receive the most appropriate and effective therapies based on their specific tumor profiles. In keeping with these needs, measurements of exosomal LINE‐1 mRNAs may provide a reliable source of biological material to accurately evaluate the clinicopathological profiles of patients with NSCLC. ROC analyses of LUSC and LUAD patients showed that exosomal LINE‐1 mRNA levels differentiated between cancer stages, with training cohort AUC values ranging from 0.863 to 0.980 for females and 0.871 to 0.957 for males, and validation cohort AUC values ranging from 0.871 to 0.957 for females and 0.853 to 0.957 for males. Comparable patterns were observed when examining tumor sizes, lymph node metastasis, and disseminated metastases.

Several studies in the recent past examined the potential clinical utility of noninvasive liquid biopsies in the diagnosis of NSCLC, with emphasis focused on discrimination between cancer and healthy individuals. Use of different biomarkers including circulating tumor DNA, circulating tumor cells, and DNA methylation has demonstrated comparable diagnostic accuracies for early‐stage NSCLCs, with AUCs ranging from 0.84 to 0.87 [44]. Lung cancer‐associated T cell repertoire in noninvasive cancers has also been employed for early detection of Stage I lung cancer with an AUC of 0.91 (SEN and SPE were 72 and 91, respectively) [45]. Also, a 10‐plasma EV circRNA signature distinguished NSCLC patients from Cont. with an AUC of 0.86 [46]. The methylation patterns in cell‐free DNA also serve as a diagnostic biomarker for early lung cancer with an AUC of 0.956 and SEN of 66.3 [47]. IGLV1‐40 and IGHV4‐4 plasma‐derived exosomal immunoglobulins were recently used to diagnose NSCLC with an AUC of 0.83 for IGHV4‐4, 0.88 for IGLV1‐40 and 0.93 for the combination [48]. Moreover, liquid biopsies using eight protein tumor markers (CA125, CA15.3, CEA, CYFRA 21‐1, HE4, NSE, proGRP, and SCCA) could detect lung cancer, NSCLC, and SCLC with 46, 25, and 40% SEN, respectively. Multiparametric models combining tumor markers and circulating tumor DNA enhanced SEN to 65, 67, and 50% [49]. To the best of our knowledge, however, our study is the first to examine the utility of exosomal LINE‐1 mRNAs in a liquid biopsy to discriminate the two main NSCLC histologic subtypes and to investigate the profound sex differences that characterize NSCLC and their clinicopathologic profiles. The high SEN and SPE of LINE‐1 mRNA cargos in exosomes indicate that these analytes may provide exquisite clinical utility as a minimally invasive biomarker of NSCLC for use in the diagnostic and prognostic evaluation of this patient population.

Our study had several limitations including the relatively small number of samples and the lack of molecular profiling of driver tumor mutations in the tumors, including TP53, EFGR, and KRAS. Further, the possibility remains that molecular heterogeneity among the NSCLC patients examined contributed to the dispersion of values seen in some cases. These limitations notwithstanding, the evidence presented here establishes with confidence the diagnostic and prognostic utility of LINE‐1 analytes in plasma exosomes to evaluate patients with NSCLC. Differential levels of LINE‐1 mRNAs correlated tightly with clinicopathological characteristics and showed high SEN and SPE in differentiating cases from Cont., histologic subtypes, and sex‐specific profiles.

In summary, our findings highlight exosomal LINE‐1 mRNA as a readily accessible biomarker for precision profiling of NSCLC. The strong diagnostic and prognostic performance of exosomal LINE‐1 mRNA as a liquid biopsy underscores its potential to improve the clinical management of patients with NSCLC. Measurements of ORF1 and ORF2 mRNAs demonstrated high sensitivity and specificity across all cancer stages in both female and male patients, with expression patterns correlating with histologic subtype, tumor stage, tumor size, lymph node involvement, distant metastasis, and smoking status. We conclude that circulating exosomes represent a reliable and minimally invasive biospecimen for precision profiling that can be developed into a novel liquid biopsy platform to advance the clinical care of patients with NSCLC.

## 4 Material and Methods

### 4.1 Plasma Samples and Patient Records

All plasma samples were deidentified and secured following informed consent from all participants per IRB approval. The collection and distribution of samples were conducted in compliance with established ethical standards. In the training cohort, a total of 186 human plasma samples stored in K2EDTA or NaCit vacutainer tubes were examined. Sixty‐two ostensibly healthy Cont. subjects were compared with 62 LUSC subjects or 62 LUAD subjects. Cont. samples were procured from BioIVT (Westbury, NY, USA) while NSCLC samples were from both BioIVT and Precision for Medicine (Norton, MA, USA). The validation cohort consisted of 114 samples obtained from both BioIVT and Precision for Medicine and grouped as follows: 38 control subjects, 38 LUSC subjects, and 38 LUAD subjects. The training cohort was constituted as follows: sex (both females and males), age (67.17 ± 1.45 for control, 67.99 ± 1.54 for LUSC, and 67.3 ± 1.4 for LUAD). The validation set was constituted as follows: sex (both females and males), age (69.12 ± 0.84 for control, 70.34 ± 1.35 for LUSC, and 70.35 ± 1.37 for LUAD). Designations of smoking histories (categorized as never smokers, former smokers, and current smokers) and staging based on the American Joint Committee on Cancer TNM system [50] were comparable for both cohorts. The clinical and demographic profiles of Cont., LUSC, or LUAD subjects of these cohorts are summarized in Table 3. All measurements were stratified by sex based on prior reports from our group suggesting sex‐specific differences in the transgenic mouse model of LINE‐1 activity [51].

**TABLE 3:** Clinical profiles of subjects in the training and validation cohorts.

		Training cohort			Validation cohort		
			NSCLC			NSCLC	
Clinical profile		Cont. (*N* = 62)	LUSC (*N* = 62)	LUAD (*N* = 62)	Cont. (*N* = 38)	LUSC (*N* = 38)	LUAD (*N* = 38)
Gender, *N* (%)	Male	31 (50)	31 (50)	31 (50)	19 (50)	19 (50)	19 (50)
	Female	31 (50)	31 (50)	31 (50)	19 (50)	19 (50)	19 (50)
Age (mean ± SE)		67.17 ± 1.45	67.99 ± 1.54	67.3 ± 1.4	69.12 ± 0.84	70.34 ± 1.35	70.35 ± 1.37
Smoking history, *N* (%)	Never	N/A	21 (33.87)	31 (50)	N/A	8 (21.05)	13 (34.21)
	Former	N/A	28 (45.16)	23 (37.1)	N/A	17 (44.74)	17 (44.74)
	Current	N/A	13 (20.97)	7 (11.29)	N/A	11 (28.95)	7 (18.42)
	N/A	N/A	−	1 (1.61)	N/A	2 (5.26)	1 (2.63)
**TNM staging**							
Cancer stage, *N* (%)	I–II	−	29 (46.77)	27 (43.55)	−	17 (44.74)	18 (47.37)
	III–IV	−	29 (46.77)	31 (50)	−	18 (47.37)	19 (50)
	N/A	−	4 (6.45)	4 (6.45)	−	3 (7.89)	1 (2.63)
Tumor size (*T*), *N* (%)	*T* < 3	−	37 (59.68)	45(72.58)	−	20 (52.63)	30 (78.95)
	*T* ≥ 3	−	23 (37.10)	11(17.74)	−	15 (39.47)	6 (15.79)
	N/A	−	2 (3.13)	6 (9.68)	−	3 (7.89)	2 (5.26)
Lymph node involvement, *N* (%)	No	−	27 (43.55)	28 (45.16)	−	16 (42.11)	11 (28.95)
	Yes	−	34 (54.84)	30 (48.39)	−	19 (50)	25 (65.79)
	N/A	−	1 (1.61)	4 (6.45)	−	3 (7.89)	2 (5.26)
Disseminated metastases, *N* (%)	Nonmetastatic	−	49 (79.03)	42 (67.74)	−	32 (84.21)	25 (65.79)
	Metastatic	−	11 (17.74)	16 (25.81)	−	3 (7.89)	10 (26.32)
	N/A	−	2 (3,23)	4 (6.45)	−	3 (7.89)	3 (7.89)
**Radiation**							
Treatment, *N* (%)	No radiation	−	12 (19.35)	12 (19.35)	−	4 (10.53)	1 (2.63)
	Radiation	−	9 (14.52)	6 (9.68)	−	6 (15.79)	5 (13.16)
	N/A	−	41 (66.13)	44 (70.97)	−	28 (73.68)	32 (84.21)

Abbreviations: Cont., control; LUSC, lung squamous cell carcinoma; LUAD, lung adenocarcinoma; NSCLC, non‐small cell lung cancer; NA, not available.

### 4.2 Isolation and Characterization of Exosomes From Cell‐Free Plasma Samples

One milliliter of the sample was centrifuged for 10 min at 4°C and 16,000×*g* to remove residual cells, cellular debris, apoptotic bodies, and nuclei. The supernatant was transferred to a new tube without disturbing the pellet, and the exosomes within the supernatant were extracted using the exoEasy Maxi Kit (Qiagen, catalog number: 76064) in accordance with the manufacturer's instructions. The exoEasy Maxi Kit isolates exosomes using membrane‐affinity spin column technology, which selectively binds exosomes while washing away contaminants and provides reproducible and efficient exosome recovery suitable for clinical biomarker studies. Although minimal risk exists of coisolation of nonexosomal particles, enrichment was validated to ensure high exosome purity. Western blot analysis was used to confirm the presence of exosomes by identifying exosomal marker proteins. Exosomes from individual sample were eluted in 400 µL of buffer and characterized using the Nanosight NS300 instrument (NTA) (Nanosight, USA). For each sample, 100 µL of exosomes was used for RNA extraction.

### 4.3 Exosomal LINE‐1 ORF1 and ORF2 mRNAs Analyses by Realtime‐PCR

Phase separation and the RNeasy Mini Kit (Qiagen, catalog number: 74106) were used to extract exosomal RNAs. To each tube, 500 mL of Trizol LS Reagent and 200 mL of chloroform were added. For phase separation, the samples were centrifuged at 12,000×*g* for 15 min at 4°C after being mixed for a minimum of 30 s and allowed to sit at room temperature for 10 min. The aqueous top layer was removed and sodium acetate [3 M, pH 5.5] (0.1 volume), RLT buffer (3.5 volume), and absolute ethanol (2.5 volume) added to the mix. The RNeasy Mini Kit was used to extract RNA and final concentrations were measured using Gen 5 data analysis software (Bioteck, USA). A total of 100 ng of each RNA sample was used to synthesize cDNA using Reverse Transcription System (Promega, USA). cDNA samples were then subjected to RT‐qPCR using Power SYBR Green Master Mix (Applied Biosystems, USA) and primer sets specific for reference genes (18S rRNA), LINE‐1 ORF1, and LINE‐1 ORF2 mRNAs (Table 4). Following these conditions: initial denaturation at 95°C for 10 min, 40 cycles of denaturation at 95°C for 15 s, and 1 min of annealing at 60°C, the RT‐qPCR reaction was carried out using the CFX96 Touch Real‐Time PCR Detection System (Biorad, USA). The 2^^∆∆Ct^ method, where Ct is the threshold cycle, was used for calculating the fold changes.

**TABLE 4:** Primer sequences of the targeted gene.

Primers	Sequence (5′–3′)
LINE‐1‐ORF1‐Fw	5′‐AGAACGCCACAAAGATACTCCTCG‐3′
LINE‐1‐ORF1‐Rv	5′‐CTCTCTTCTGGCTTGTAGGGTTTCTG‐3′
LINE‐1‐ORF2‐Fw	5′‐AAACTGAACAACCTGCTCCTGAATG‐3′
LINE‐1‐ORF2‐Rv	5′‐CTACACACTGCTTTGAATGCGTCC‐3′
18s rRNA‐Fw	5′‐TGCCCTATCAACTTTCGATGGTAGTC‐3′
18s rRNA‐Rv	5′‐TTGGATGTGGTAGCCGTTTCTCA‐3′

### 4.4 ROC Analyses

ROC curve analysis was used to evaluate the performance of a binary classification method. The analyses included assessments of AUC, SPE, and SEN calculations to evaluate the diagnostic and prognostic value of plasma exosomal LINE‐1 analytes in NSCLC. Logistic regression was used to compare each pair of AUCs. The guidelines developed by Ref. [52] were employed to assess the adequacy of model discrimination using AUC measures analysis as follow; AUC = 0.50 indicated no model discrimination, poor discrimination for 0.50 < AUC < 0.70, acceptable discrimination for 0.70 < AUC < 0.80, excellent discrimination for 0.80 < AUC < 0.90, and superior discrimination for AUC > 0.90. A *p* < 0.05 was defined as statistically significant.

### 4.5 Statistical Analysis

GraphPad Prism, version 9.5.0 (GraphPad Software, San Diego, CA, USA) was used for statistical analyses. ANOVA and Tukey's HSD test were utilized to estimate differences in plasma exosomal LINE‐1 levels between LUSC, LUAD patients, and ostensibly healthy Cont. Unpaired *t*‐test analysis was used to compare the levels of LINE‐1 between subgroups and Grubbs’ test to identify outliers. Significant outliers at *p* < 0.05 were excluded from statistical analysis. In all analyses, *p* < 0.05 was considered significant, and *p* < 0.01 was considered highly significant.

## Author Contributions

A.A.I.H. designed the research, performed experiments, analyzed data, interpreted results, prepared figures, and drafted the manuscript. K.S.R. conceived the project, designed the research, analyzed data, interpreted results, and edited the manuscript. All authors have read and approved the final submission.

## Funding

This work was supported in part by NIH grant ES034542, a Governor's University Research Initiative and CPRIT grant RP230204 to K.S.R. and the Arab Fund Fellowship Program (The Distinguished Scholar Award) to A.H.

## Ethics Statement

The plasma samples used in this study were obtained from a certified commercial vendor as deidentified specimens. Institutional Review Board (IRB) approval (Biovit Protocol Numbers; AST‐FPB‐005, AST‐FPB‐US, AST‐FPB‐005‐VN, AST‐FPB‐nonUS, AST‐FPB‐nonUS‐Sof, 05035, Sera Trials – 11008, Sera Trials‐11004, 12020; Precision for medicine Protocol Number, PFM064) documented that informed consent was obtained from all donors and that sample collection and distribution complied with ethical regulations and standards.

## Conflicts of Interest

Kenneth S. Ramos is an editorial board member of MedComm. He was not involved in the review of this submission nor decisions related to this manuscript. Abeer A.I. Hassanin declares no conflicts of interest.

## Data Availability

The datasets used and/or analyzed during the current study are available from the corresponding author upon request.
