# Correction to “Exosomal Long Interspersed Nuclear Element‐1 Analytes Discriminate Histologic Subtypes, Sex, and Clinicopathological Characteristics of Patients With Non‐Small Cell Lung Cancer”

**DOI:** 10.1002/mco2.70565

**Published:** 2026-01-04

A. A. I. Hassanin and K. S. Ramos, “Exosomal Long Interspersed Nuclear Element‐1 Analytes Discriminate Histologic Subtypes, Sex, and Clinicopathological Characteristics of Patients With Non‐small Cell Lung Cancer,” *MedComm* 6, no. 11 (2025):e70472. https://doi.org/10.1002/mco2.70472.

1. In the title of the paper, the text “Long Interspersed Nuclear Element‐1” was incorrect. This should have read as “Long Interspersed Element‐1.”
2. In the *Abstract* section, the text “Long Interspersed Nuclear Element‐1” was incorrect. This should have read as “Long Interspersed Element‐1.”
3. In paragraph 3 of the *Introduction* section, the text “Long Interspersed Nuclear Element‐1” was incorrect. This should have read as “Long Interspersed Element‐1.”

The authors apologize for this error.

